# Dual Roles of the Activin Signaling Pathway in Pancreatic Cancer

**DOI:** 10.3390/biomedicines9070821

**Published:** 2021-07-14

**Authors:** Wanglong Qiu, Chia-Yu Kuo, Yu Tian, Gloria H. Su

**Affiliations:** 1 The Department of Pathology and Cell Biology, Columbia University Irving Medical Center, New York, NY 10032, USA; wq2102@columbia.edu (W.Q.); charleskuo0401@gmail.com (C.K.); ytian2450@163.com (Y.T.); 2 Herbert Irving Comprehensive Cancer Center, Columbia University Irving Medical Center, New York, NY 10032, USA; 3 Department of Otolaryngology and Head and Neck Surgery, Columbia University Irving Medical Center, New York, NY 10032, USA

**Keywords:** activin signaling pathway, ACVR1B (Activin A Receptor Type 1B), pancreatic cancer, PDAC (pancreatic ductal adenocarcinoma), tumor suppression, context-dependent, TGFβ superfamily, therapeutic target

## Abstract

Activin, a member of the TGF-β superfamily, is involved in many physiological processes, such as embryonic development and follicle development, as well as in multiple human diseases including cancer. Genetic mutations in the activin signaling pathway have been reported in many cancer types, indicating that activin signaling plays a critical role in tumorigenesis. Recent evidence reveals that activin signaling may function as a tumor-suppressor in tumor initiation, and a promoter in the later progression and metastasis of tumors. This article reviews many aspects of activin, including the signaling cascade of activin, activin-related proteins, and its role in tumorigenesis, particularly in pancreatic cancer development. The mechanisms regulating its dual roles in tumorigenesis remain to be elucidated. Further understanding of the activin signaling pathway may identify potential therapeutic targets for human cancers and other diseases.

## 1. Introduction

Activins are growth factors of the transforming growth factor-β (TGF-β) superfamily. The TGF-β superfamily proteins are not only involved in embryonic development and skin morphogenesis, but also hold the key to the development of many human diseases, including cancers. Activin shares similar structures with other members of the TGF-β superfamily proteins and has a parallel signaling pathway to transduce signals from the extracellular compartment into the nucleus. The downstream effects of the activated TGF-β superfamily signaling pathways lead to switching on or off the expression of the target genes to regulate cellular responses [1].

In comparison to the TGF-β and bone morphogenesis (BMPs) pathways in the TGF-β superfamily, activin signaling is less well understood. An aberrant activin signaling pathway has been found to be associated with several disease conditions, including preterm labor with delivery, osteoporosis, cancer, and cancer-related cachexia [2,3,4,5,6]. This review focuses on the activin signaling pathway and its role in tumorigenesis, especially pancreatic cancer.

## 2. Activin and the Regulation of the Activin Signaling Pathway

### 2.1. Components of the Activin Signaling Pathway

Activin was first discovered from the purification of inhibin and was found to have an opposing ability to inhibin in regulating the secretion of pituitary follicle-stimulating hormone (FSH) in the anterior pituitary [7]. Inhibins are formed from heterodimers of inhibin α and inhibin β subunits: inhibin A (α: β_A_) and inhibin B (α: β_B_). Activin is a dimeric protein of two inhibin β-subunits, and the dimeric structure of activin is maintained by a single disulphide bond between the two subunits [8]. There are currently four known inhibin β subunits (β_A_, β_B_, β_C_, and β_E_), which can form five types of activin proteins, activin A (β_A_: β_A_), B (β_B_: β_B_), C (β_C_: β_C_), E (β_E_: β_E_), and AB (β_A_: β_B_), through homo- or hetero-dimerization [9]. A-E β-subunits are expressed in different tissues and organisms [10,11,12]. The biological activities of activins are mediated by heteromeric receptor complexes consisting of two different types of receptors: type I and type II. Activin binds to one of the two type II receptors ACVR2A (Activin A Receptor Type 2A) or ACVR2B (Activin A Receptor Type 2B), which prompts the recruitment of a type I receptor, such as ACVR1B (Activin A Receptor Type 1B, also known as activin-like kinase 4, ALK4). Subsequently, the activated ACVR1B/ALK4 receptor recruits and phosphorylates SMAD2 (SMAD Family Member 2) and SMAD3 (SMAD Family Member 3), which then bind to the coregulatory SMAD4 (SMAD Family Member 4), and the complexes translocate into the nucleus where they regulate gene transcriptions [13,14] (Table 1).

### 2.2. Regulation of the Activin Signaling Pathway

Three type I receptors (ACVR1, ACVR1B, and ACVR1C) have been identified for activin signaling: ACVR1 (Activin A Receptor Type 1, also known as ALK2) primarily participates in the BMP signaling pathway [15]; ACVR1B/ALK4 is the major type I receptor for the activin signaling pathway; while ACVR1C (Activin A Receptor Type 1C, also known as ALK7) mainly takes part in the nodal signaling pathway [16]. When activated by ligands, activin type II receptors recruit type I receptors and phosphorylate the GS-box on the cytoplasmic domain of the activin type I receptors. The phosphorylated GS-box, in succession, unblocks and releases the kinase activity of the activin type I receptor kinase domain [17,18]. The open active cleft of the activin type I receptor kinase domain can then phosphorylate receptor-regulated SMAD (R-SMAD) by its kinase activity. Distinct R-SMADs are recruited in different TGF-β family signaling pathways: SMAD2 and SMAD3 are involved in the TGF-β and activin signaling pathways, while SMAD1, SMAD5, and SMAD8 mediate BMP signaling. Once the signaling pathway is activated, dephosphorylation of these signaling transducers is a key mechanism to regulate signaling transduction. Protein phosphatase PPM1A, which was identified to negatively regulate the activin signaling pathway, can dephosphorylate phospho-SMAD2 and enable SMAD2 to relocate back to the cytoplasm and terminate signal transduction [19].

**Figure 1:**
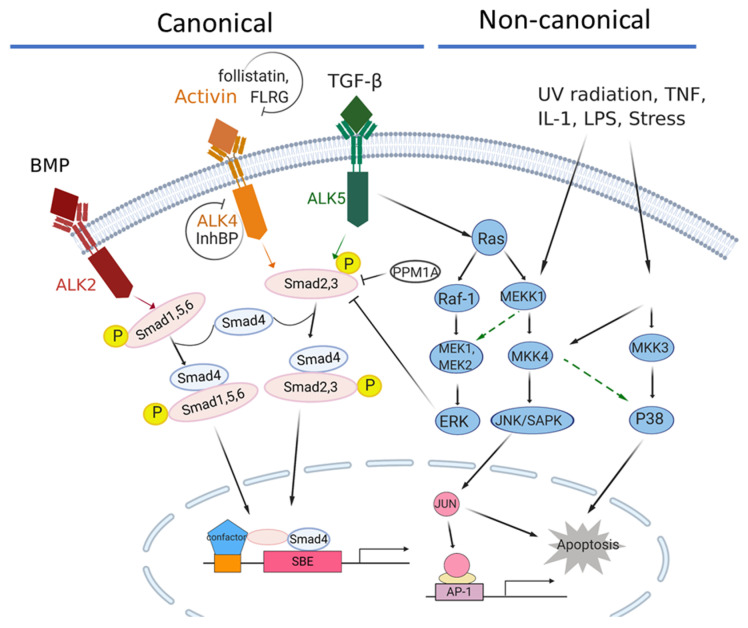
The activin/TGFβ signaling network in pancreatic cancer. The canonical signaling axis of the TGF-β superfamily members is Smad4-dependent, while the non-canonical signaling is Smad4-independent. ACVR1B/ALK4 is the major type I receptor for the activin signaling pathway, while TGFRB1/ALK5 and ACVR1/ALK2 are the major type I receptors for TGFβ and BMP signaling, respectively. In the canonical activin signaling, the pathway is activated by the binding of activins to one of the two type II receptors, ACVR2A or ACVR2B, which then recruit a type 1 receptor, such as ACVR1B/ALK4. The activated ACVR1B/ALK4 receptor subsequently recruits and phosphorylates SMAD2 and SMAD3, which then bind to the coregulatory SMAD4, and the complex translocates into the nucleus where it regulates gene transcriptions. Activin bioactivity can be modulated by antagonists, such as Follistatin, FLRG (follistatin-related gene), InhBP (inhibin-binding protein), etc.

Activin bioactivity can be further regulated by various extracellular antagonists such as inhibins, follistatin, follistatin-related gene (FLRG) [20] and by membrane co-receptors such as Cripto or BAMBI [21,22]. Whereas structurally-related inhibin antagonizes the action of activin by competing against activin for the activin type II receptors, follistatin and FLRG directly bind to activin with high affinity, thereby preventing activin from binding to its membrane receptors [23,24,25,26]. The β-subunit of inhibin can bind to activin type II receptors such as activin, but the α-subunit of inhibin is unable to bind and recruit ACVR1B [27,28,29]. However, a cellular membrane co-receptor betaglycan, which has high affinity for inhibin [30], can enhance the ability of inhibin to antagonize activin activity by forming the complex of inhibin, betaglycan and activin type II receptors [31]. Another inhibin co-receptor, inhibin-binding protein or InhBP, has been shown to be associated with ACVR1B/ALK4 but not activin type II receptors. The complexes of inhibin and InhBP can disrupt the binding of ACVR1B to activin type II receptors (Chapman and Woodruff, 2001). These inhibin co-receptors regulate ligand binding and the formation of activin receptor complexes. Follistatin and FLRG modulate activin signaling transduction by two major mechanisms. First, follistatin can compete with activin receptors through direct binding to activin in circulation or at the cell membrane and block the formation of ligand and receptor complexes [32]. Similarly, FLRG can also antagonize activin by binding to activin type II receptors [33]. Alternatively, the membrane-bound follistatin-288 can internalize activin by endocytosis and lead to the lysosomal degradation of activin [34,35]. Thus, activin signaling can be regulated at the levels of ligand binding, receptor complex formation, or the activation of downstream mediators (Table 1 and Figure 1).

## 3. Functions of Activin Signaling

### 3.1. Embryogenesis and Hair Follicle Development

During mammalian development, activin has critical roles in the development of face, whiskers, hair follicles, heart, and digestive tract, which all involve epithelial–mesenchymal interactions [36]. Conventional activin βA-subunit-deficient mice have no whiskers or incisors and have defects in mandible, secondary palate, molars, and eyelids. These knockout mice died within 24 h of birth [2]. Activin-βB-deficient mice were viable but with defects in eyelid formation; female mice harbored additional flaws in ovary formation. The compounded phenotypes of mice with double deletion of both βA- and βB-subunits revealed that these two subunits have distinct functions in development [2]. Mutation in one of the activin type II receptors led to similar developmental defects as those observed in activin-βA-deficient mice [37]. Fetuses that lacked both activin type II receptors died in utero. Mutants with deficiency in activin βA, activin type II receptors, or Smad2 shared similar tooth malformation [38]. All of these data have unequivocally demonstrated the importance of the activin signaling pathway in mammalian development.

Activin and follistatin serve as regulators of hair follicles in utero and beyond [36]. In hair follicle development, activin-βA produced by mesenchymal cells can modulate epithelial cells of hair follicles by binding to activin receptors and inhibiting the development of hair follicles. In contrast, follistatin produced by epithelial cells can bind to activin and counteract the inhibitory effects of activin on hair follicles [39]. Dominant-negative *Acvr1b* transgenic mice exhibited delays in the early stage of hair follicle development [40]. Conditional deletion of *Acvr1b* in mouse skin facilitated by K14-Cre resulted in hair loss through failure in hair cycle reentry after morphogenesis [41]. The phenotypes of activin-βA-deficient mice and dominant-negative *Acvr1b* transgenic mice reveal that activin is required for vibrissae follicle development but is not essential for pelage hair follicle development [37,40]. Intriguingly, overexpression of activin-βA in mice led to similar phenotypes as those observed in the dominant-negative *Acvr1b* transgenic mice, which exhibited hair follicle recycling delay during catagen [39,40]. Moreover, the overexpression of activin-βA can lead to down-regulation of Bmp2 and up-regulation of the Bmp2 inhibitor [39], which are also important in hair follicle recycling [42].

In addition to its importance in hair follicle development and recycling, animal studies using genetically engineered mice also revealed the importance of activin signaling in skin morphogenesis. Follistatin knockout pups developed shiny and taut skin due to hyperactive keratinocytes [43], suggesting that a high concentration of activin has an effect on keratinocytes [36]. However, overexpression of follistatin or dominant-negative *Acvr1b* transgenic mice display phenotypically normal proliferation and differentiation of keratinocytes [40,44]. The low activin in keratinocytes of adult skin has no significant effect in proliferation and differentiation, which might be compensated for by TGF-β, while embryonic and newborn skin would be more susceptible to dysregulated expression of activin. Activin secreted by dermis fibroblasts tends to inhibit proliferation of keratinocytes, but follistatin produced by keratinocytes can counterblock this suppression by binding to activin. These interactions between activin and follistatin play a key role in wound healing; hence, mice with overexpression of follistatin have problems with delayed scar formation, closure of wounds, and small scar areas, but mice with overexpression of activin-βA have faster wound repair capability [44].

Activin also controls cellular growth and developmental differentiation of many other cell types [45,46]. For instance, activin βB knockout mice developed defective ductal glands and lobuloalveolar buds [47], suggesting that activin is a critical regulator in mammary gland development [48]. Activin βA deficient mice died before mammary gland development due to palate malformations [2]. Dysregulation in activin signaling can also result in abnormalities in female reproduction capabilities [49,50]. Activin receptor signaling is required for the majority of myelin generation in development and following injury, and dysregulated activin receptor signaling contributes to diseases of myelin disorders [51]. Activin signaling has multifaceted activities throughout mammalian development.

### 3.2. Cell Proliferation, Cell Cycle Arrest, and Apoptosis

Similar to the TGF-β signaling pathway, activation of the activin signaling axis is anti-proliferative in certain cell types [52]. Activin, by stimulating the expressions of p21 and p27 CDK inhibitors, is anti-proliferative and induces G1 phase cell cycle arrest, which are mediated with the involvement of pRb, cyclins, CDKs, and/or p53 as reported previously [45,53,54,55,56,57,58,59]. Activin can also induce programmed cell death, namely apoptosis, as an alternative means to restrain cell growth. Activin treatment was found to increase apoptosis of LNCaP cells by up-regulating p53 and down-regulating anti-apoptotic protein Bcl-2 [60]. In hematopoietic cells, activin-mediated apoptosis was dependent on SMAD-induced expression of inositol phosphatase SHIP (Src homology 2 domain-containing inositol phosphatase). Activin/SMAD-induced SHIP expression resulted in altered phospholipid metabolism, the inhibition of Akt/PKB phosphorylation, and cell death [61]. Activin-mediated arrest of cell proliferation and cell cycle through non-SMAD signaling pathways, such as the PI3K/AKT and MARK/p38/JNK pathways, have also been reported [59,62].

### 3.3. Tumor Suppression

Canonical activin signaling shares the same SMAD mediators with TGF-β, which is a well-known inhibitor of epithelial cell proliferation. These SMAD mediators are also known to regulate tumorigenesis in many aspects such as cell proliferation, cell cycle, and apoptosis. Therefore, perhaps it not a surprise that activin is deemed antitumorigenic and can inhibit proliferation and stimulate apoptosis in cancer cells as described above [52,58,59,60,61]. Consistent with this notion, inactivating genetic mutations of various components within the activin signaling pathway have been reported in cancers, presumably as a common mechanism for tumor cells to escape activin-mediated growth inhibition. Somatic biallelic inactivation of *ACVR1B* has been reported in pancreatic cancer [63,64]. Two 8-bp polyadenine tracts of the *ACVR2* gene are frequent targets of inactivating frameshift mutations in gastrointestinal tumors with microsatellite instability (MSI) [65,66,67]. In addition, biallelic inactivation of *ACVR2* in non-MSI cancer cells has also been reported for prostate cancer [67]. The truncated ACVR2 protein resulting from the frameshift mutation has been demonstrated to have significant functional deficiency on activin signaling transduction [67]. Restoration of ACVR2 expression in MSI^+^ colorectal cancers with *ACVR2* mutation exhibited growth inhibition but increased cell migration [68]. This biological evidence in support of the genetic studies, further cemented the tumor-suppressive role of activin signaling in tumorigenesis. Mutational inactivation of downstream mediators of the activin signaling axis that overlap with TGF-β, such as the SMAD2/3/4 [69,70,71,72,73,74], has been well-documented in many cancer types, providing further genetic support that activin/TGF-β signaling axes are tumor-suppressive and are selectively inactivated genetically during tumor clonal evolution.

Epigenetic dysregulation of the activin signaling pathway has also been described and is more prevalent among human cancers than are the genetic events. Downregulated expressions of activin type II receptors ACVR1B, and SMAD4 were observed in ER-negative breast cancer cell lines [75]. Activin inhibitors, both follistatin and FLRG, were overexpressed in carcinoma compared to that of adjacent normal tissues in breast cancer and hepatocellular carcinoma [76,77,78]. Follistatin was identified as one of the bone metastasis signature genes in breast cancer [79]. Silencing of FLRG by siRNA inhibited the cell growth of human breast tumors [33].

Biologically, activin-induced growth suppression and cell death were shown to be dependent on p21 in colon cancer [80]. It has also been demonstrated that activin could exert its tumor suppression via the inhibition of telomerase activity and the expression of the *hTERT* gene in human breast cancer and cervical cancer cell lines [81]. Telomerase activity is one of the key mechanisms that are often hijacked by tumor cells to gain immortality. Because concomitant increase of SMAD3 activation and decrease of the hTERT promoter activity were observed, it was postulated that activin-induced repression of the *hTERT* gene was mediated by SMAD3-dependent regulation of the *hTERT* promoter activity in this context [81].

Alternative mechanisms may also be involved in activin-induced tumor-suppression. For instance, activin can increase the expression of a neural cell adhesion molecule (NCAM), and NCAM was found to attenuate tumor cell invasiveness in human breast cancer cell lines [82,83]. However, a direct link between activin and NCAM remains to be established. Angiogenesis is another feature of tumorigenesis that may also be regulated by activin signaling. Angiogenesis is not only important for supplying nutrients to tumor cells, but also a means by which tumor cells can metastasize. Treating neuroblastoma xenografts with activin A led to reduced tumor growth and decreased vascularity of the xenografted tumors. It was proposed that activin inhibited the growth of vascular endothelial cells by reducing the expression of vascular endothelial growth factor receptor-2, which is an important receptor for angiogenesis [84,85].

Given the paramount genetic and biological evidence, it is unequivocal that the activin signaling pathway possesses tumor-suppressive functions in cancer development and progression. However, similar to TGF-β signaling, which is well-known for its dual role in tumor promotion and suppression [1], activin signaling can be exploited by cancer cells for their growth advantage paradoxically, despite its evident role in tumor suppression.

### 3.4. Tumor Progression and Metastasis

Overexpression of activin A was detected in the majority of the patients with granulosa cell tumors [86] and almost all of the ovarian mucinous carcinomas except epithelial tumors [87], esophageal [88], and colorectal carcinomas [89,90]. Expressions of activins and components of its signal transduction were also detected in normal and cancer breast tissues [91,92]. The deregulation of activin signaling correlated strongly with increasing breast cancer grade [93,94]. Higher levels of activin were also detected in the sera of patients with breast cancer compared to that of the normal controls [95]. Elevated activins were also detected in breast cancer patients with metastasis or in their tumor tissues [96,97,98]. These data indicate that activin signaling may also function as an oncogene in human cancers.

Functional studies showed that activin could increase tumor growth in mammary carcinoma cells, but inhibited angiogenesis in comparison to follistatin-expressed cells [57]. Exogenous overexpression of activin in human esophageal carcinoma cell lines induced increased tumor proliferation and progressive phenotypes [99]. Consistent to this, activin was reported to enhance esophageal tumor malignancy via the upregulation of N-cadherin and MMP7 (matrix metalloproteinases 7) [99,100,101]. In hepatocellular carcinoma, activin was found to stimulate the expression of VEGF in a Sp1-dependent manner [102]. Mice with both inhibin α- and ActRIIA-deficiency developed tumors without the cancer cachexia, which had been previously reported in inhibin α-deficient mice [103].

The mechanisms by which activin signaling enhances the later stage of cancer progression are not yet conclusive. Activin signaling is capable of directly modulating the cancer cells and their malignancy. It is reported that breast cancer cells become resistant to activin inhibition as they gradually lose estrogen receptor expression and become less differentiated [75]. This growing resistance to activin may be due to the inherent increased malignancy of ER-negative breast cancer cells compared with that of ER-positive cells, or because activin and estrogen can antagonize each other’s biological activities in human breast cancer cells [104]. In the breast cancer cell lines, the conversion from non-invasive epithelial-like CD44^+^CD24^+^ cells to invasive mesenchymal CD44^+^CD24^−^ progeny was also found to be activin/nodal-dependent [105]. The epithelial-mesenchymal transition (EMT) is widely recognized as a molecular mechanism involved in the migration and metastasis of cancer cells. TGF-β is the canonical inducer of EMT in human cancers through the downregulation of cell adhesive molecule E-cadherin and upregulation of matrix metalloproteinases (MMPs). Activins were observed to induce EMT and invasion in ovarian cancer and colon cancer [106,107]. In primary colon cancer, activin induced EMT via PI3K activation in a Smad4-independent manner [107].

Moreover, activin may influence the metastatic process indirectly by altering the tumor microenvironment (TME). It was shown previously that the switch of TGF-β in breast cancer from a tumor-suppressive role to a tumor promoting one was due to the recruitment of myeloid-derived suppressor cells (MDSCs) into the TME [108]. The disruption of *Tgfbr2* signaling in cancer cells resulted in increased chemokine signals SDF-1/CXCR4 and CXCL5/CXCR2 that enhanced MDSC infiltration into tumors, which led to the promotion of tumor invasion and metastasis [108]. Activins have been shown to affect cell-mediated immunity by modulating monocyte chemotaxis, monocyte migration, and cytokine production [109,110,111]. Therefore, it is conceivable that activin signaling may play a similar role in the TME as that of TGF-β. Recently Cangkrama et al. demonstrated that activin A secreted by tumor cells can activate pro-tumorigenic cancer associated fibroblasts in non-melanoma skin cancer [112]. Bauer et al. reported that activin A secreted by stromal cells can induce migration and EMT in colorectal cancer cells [113]. In essence, activin signaling can stimulate cancer growth via its direct or indirect activities in the tumor cell compartment and involves crosstalk between tumor cells and the TME.

## 4. Targeting Activin Signaling Pathway in Pancreatic Cancer

### 4.1. Activin Signaling and the Development of the Pancreas

During mouse embryogenesis, a nascent dorsal pancreatic bud begins to develop out of the foregut endoderm around E8.5–E9.5, followed by the development of paired ventral pancreatic buds at E10. Activin signaling plays a direct inductive role in the early foregut patterning and pancreatic formation in mice [114]. Studies using knockout and transgenic mouse models have demonstrated that the activin family hormones promote commitment to the pancreatic fate and favor the development of endocrine progenitor cells [115,116,117]. Inhibition of activin signaling during embryogenesis resulted in a decreased number of endocrine progenitor cells, hypoplastic islets, and reduced differentiated β-cells that persisted into adulthood [114,117]. Studies using explant cultures or pancreatic regeneration models found that activins negatively regulated exocrine cells and favored the expansion of endocrine lineages [118,119,120], indicating that activins extend their actions from pancreatic development into regulation of adult pancreatic homeostasis. Within a regenerating adult pancreas, activins specifically inhibit the expansion of immature pancreatic cells and promote terminal differentiation [118]. It is suggested that activins may act similarly during early pancreatic tumorigenesis, promoting terminal differentiation of progenitor cells, inhibiting the expansion of the epithelial compartment, and suppressing the growth of progenitor-like tumor cells.

### 4.2. Dual Roles of Activin Signaling in Pancreatic Tumorigenesis 

**Figure 2:**
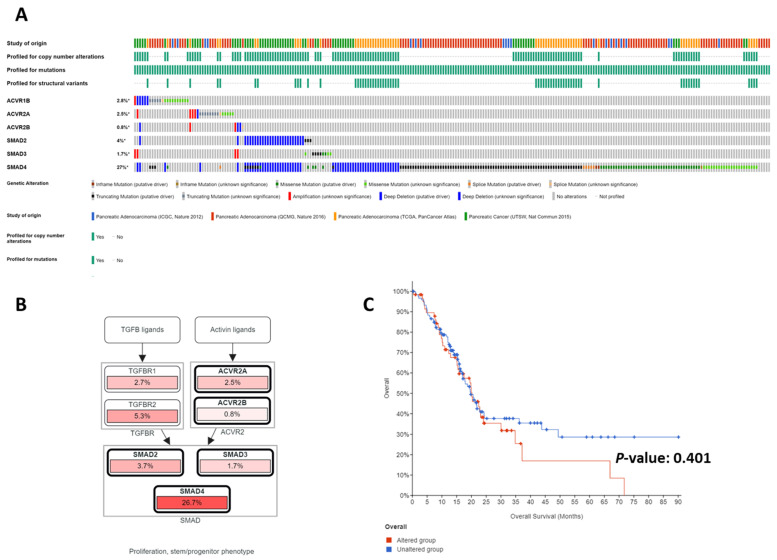
Genomic alterations in the activin/TGFβ signaling pathway. Analyses of the Cancer Genome Atlas (TCGA) databases using the cBioPortal (**A**–**C**), which revealed mostly inactivating mutations and deletions in the activin/TGFβ signaling axes (**A**). Collectively, the activin/TGFβ signaling pathway is one of the most frequently disrupted core signaling axes in pancreatic cancer (**B**). No significant correlation exists between patient survival and genomic alterations of the activin/TGFβ signaling axes, perhaps due to the dual roles of the activin/TGFβ signaling pathway in pancreatic cancer (**C**).

Genetic studies of human pancreatic ductal adenocarcinoma (PDAC) depict a tumor suppressive role for the activin signaling pathway. Both biallelic inactivation of *ACVR1B*/ALK4 and the loss of ACVR1B expression were detected in human PDAC cell lines and patient specimens [63,64] (Table 1). Biallelic inactivation of *ACVR2A* were found in MSI^+^ PDAC at a high frequency, albeit MSI^+^ PDAC are rare [66]. Biallelic inactivation of *ACVR2A* was also detected in one MSI^−^PDAC in the same study [66]. The downstream mediator of the activin/TGFβ signaling axes, *SMAD4*, is one of the most frequently inactivated tumor-suppressor genes via PDAC [69,121]. Multiple whole-genome analyses of resected PDACs confirmed that activin/TGFβ is one of the core signaling pathways frequently disrupted in PDAC (47–100%) [122,123]. Interrogations of the Cancer Genome Atlas (TCGA) databases using the cBioPortal for Cancer Genomics also revealed major mutations and deletions in the activin/TGF signaling axes (deep deletion and truncating mutations >> amplification of unknown significance) (Table 2 and Figure 2) [119,122,123], indicating that inactivating alterations in these genes (*ACVR1B*, *ACVR2A*, *ACVR2B*, *SMAD2, SMAD3, SMAD4, TGFBR1, TGFBR2*) offer selection advantages in pancreatic tumor clonal evolution. 

Consistent with the implications of the genetic evidence, we and others have shown that conditional *Acvr1b* knockout in the pancreases of mice synergized with oncogenic *Kras* induced pancreatic tumorigenesis [127,128], providing functional evidence that inactivation of the activin signaling pathway in the epithelial compartment promotes tumor development in the early stages of pancreatic tumorigenesis. Zhao et al. reported that activin A is upregulated in oncogenic Kras-induced acinar-to-ductal metaplasia (ADM) and precancerous lesions PanINs (pancreatic intraepithelial neoplasia) in mice. Intriguingly, treatment with sActRIIB-Fc to neutralize activin A was sufficient to accelerate the progression of oncogenic Kras-induced ADM to PanINs in mice [128], suggesting that upregulation of activin A and the activation of activin signaling are reactive to oncogenic Kras and serve as one of the first defense mechanisms against cell transformation to the tumorigenic state. In the absence of oncogenic *Kras*, the inactivation of *Acvr1b* alone in the pancreatic exocrine cells also led to elevated activin A levels in the pancreas, resulting in inflammation and the development of ADM and PanINs [127]. It remains to be further elucidated if under this circumstance (without an oncogene-driven initiation), the development of ADM and PanINs was a direct result of dysregulated activin signaling in the exocrine lineage or an indirect effect of an inflammatory TME. 

While the activin/nodal signaling pathway is mostly inactive in adult tissues, overexpression of activins, their receptors, and the nodal co-receptor cripto-1 have been reported in pancreatic cancer [129,130,131,132], suggesting an oncogenic role for the pathway. Systemic plasma activin levels were found to be associated with metastasis and prognosis in human pancreatic cancer [62]. Functional activation and overexpression of the components of the activin/nodal signaling pathway including *Nodal*, *Cripto-1*, *FoxH1*, *Smad2*, *Smad4*, *Gdf1*, *activin,* and *ACVR1B* were detected in primary pancreatic cancer stem cells (CSC) [133]. Since the presence of CSCs has been proposed as the major cause for chemotherapy and radiotherapy resistance, Lonardo et al. investigated if the abrogation of the activin/nodal signaling axis was sufficient to modulate the self-renewal and tumorigenicity of pancreatic CSCs. They reported that treatment with the small molecular inhibitor SB431542 against TGF type I receptors (ALK4/5/7) could mitigate the sphere formation of pancreatic CSCs in a dose-dependent manner. Using recombinant lefty as the specific endogenous nodal inhibitor or knockdown *Nodal*, *ACVR1B*, and *Smad4* also decreased the sphere formation capacity [133]. Therapeutic inhibition of the activin signaling pathway in pancreatic CSCs is potentially attractive and may be advantageous over targeting other developmental pathways (i.e., Sonic Hedgehog) because the normal pancreas and other adult tissues completely lack activin signaling activity; therefore, they may be spared from drug-related side effects [129]. Mancinelli et al. reported that pancreatic stellate cells (PSC) secreted high levels of activin A, which promoted PDAC cell migration. While treating PDAC-bearing mice with an activin A neutralizing antibody did not decrease the primary tumor burden, it significantly reduced tumor metastasis [132], further supporting a tumor-promoting role for activin A in late pancreatic tumorigenesis and the potential benefits of targeting activin signaling in preventing PDAC metastasis. 

These accumulative evidence supports a working model in which activin signaling plays a dual role in pancreatic tumorigenesis as a tumor-suppressor in the early stage and a tumor-promoter in the advanced stage. While the tumor suppressive function of the activin signaling pathway is considered to be executed in a canonical Smad-dependent manner, the tumor promoting role of activin signaling in pancreatic cancer may be via the non-Smad-dependent pathways such as MAPK/JNK and PI3K/AKT [62] (Figure 1). Perhaps because the function of the activin signaling pathway in pancreatic tumor development and progression is complex and context-dependent, the genetic alterations in the activin signaling pathway do not significantly correlate with survival in PDAC patients (Figure 2C). However, high expression of activin A in the stroma of PDAC has been reported to correlate with reduced survival for PDAC patients [132]. Therefore, it is conceivable that the stromal component also contributes to the complexity of the differential roles of activin signaling in early vs. late pancreatic tumorigenesis. 

Overexpressing follistatin in small cell lung cancer cells has proven to decrease multiple-organ metastasis in an NK cell-depleted SCID mouse model [134,135]. Novel activin antagonists, NUCC-474 and NUCC-555, identified by in silico high throughput screening have been shown to inhibit activin A-mediated cell proliferation in ex vivo ovary cultures [136]. Activin A is an autocrine activator of PSCs and abrogation of activin signaling with follistatin has been suggested as a therapeutic strategy to reduce pancreatic fibrosis [137]. Activin A expression in PDAC was found to correlate with increased cachexia severity, and systemic blockade of activin signaling could preserve muscle and prolong survival in mice [138]. As described above, a pan-TGF type I receptor inhibitor, SB431542, was effective in mitigating the sphere formation of pancreatic CSCs in a dose-dependent manner [133]. Neutralizing activin A secretion by PSCs significantly reduced tumor metastasis in a PDAC mouse model [132]. Together, these data suggest that activin antagonists may have therapeutic value in advanced pancreatic cancer (i.e., metastatic PDAC). However, given the role of activin signaling in pancreatic regeneration and homeostasis [118] and its unequivocal tumor-suppressive function in early tumorigenesis [127,128], negative regulation of activins may lead to unintended consequences and expansion of epithelial cells and/or precancerous lesions in the pancreas. Therefore, the efficacy of these inhibitors should be comprehensively evaluated with abundant caution in preclinical studies.

## 5. Conclusions

A multitude of genomic studies have shown that activin/TGFβ is one of the core signaling pathways frequently disrupted in PDAC. Increasing evidence supports that, similar to TGF-β, activin signaling plays a tumor-suppressive role in the early stage of pancreatic tumorigenesis, but switches to be a promoter in invasive pancreatic cancer. It has been challenging to dissect the complex networks involving multiple protein interactions and numerous cross-talking pathways, such as the activin signaling pathway described here, in tumorigenesis or other cellular processes. However, as emerging techniques become available, it is hoped that we will be able to unveil the complexity of the activin signaling pathway gradually, to differentiate its tumor-suppressive capabilities from its tumor-promoting functions. Personalized medicine represents the future of medicine where ligands, receptors, and mediators of activin signaling have the potential of becoming feasible targets for future therapies.

## Figures and Tables

**Table 1:** Components and modulators of the activin signaling pathway.

Pathway Components	Signaling Mediators	Antagonists
Ligands	Activin A	Inhibins A and B
	Activin B	Follistatin
	Activin C	FLRG
	Activin E	
	Activin AB	
Type II receptors	ACVR2A	Cripto
	ACVR2B	BAMBI
		Betaglycan
Type I receptors	ACVR1	InhBP
	ACVR1B	
	ACVR1C	
R-Smad	SMAD2	
	SMAD3	
Co-Smad	SMAD4	

**Table 2:** Genomic alterations of the activin/TGFβ signaling axes in PDAC.

Gene	Gene Locations	Frequency (%)	Alterations
Mutation Frequency Reported in Publications			
*ACVR1B/ALK4*	12q13.13	2–21	Mutation and deletion [63,64]
*ACVR2A/ACVR2*	2q22.3–q23.1	4 (86%/MSI^+^)	Mutation and deletion [66]
*SMAD4/DPC4*	18q21	32–55	Mutation and deletion [69,121]
*TGFBR1/ALK5*	9q22.33	1	Deletion [124]
MSI^−^/*TGFBR2*	3p24.1	3–5	Deletion [121,124]
MSI^+^/*TGFBR2*	3p24.1	3	Mutation [124]
**Mutation frequency available in The Cancer Genome Atlas (TCGA) via the cBioPortal** [125,126]			
*ACVR1B/ALK4*	12q13.13	2.8	Mutation > deletion > amplification
*ACVR2A/ACVR2*	2q22.3–q23.1	2.5	Mutation > amplification > deletion
*ACVR2B/ACTRIIB*	3p22.2	0.8	Deletion > amplification > mutation
*SMAD4/DPC4*	18q21.2	27	Mutation > deletion
*SMAD2*	18q21.1	4	Deletion > mutation
*SMAD3*	15q22.33	1.7	Mutation > amplification
*TGFBR1/ALK5*	9q22.33	2.7	Mutation > deletion > amplification
*TGFBR2*	3p24.1	5.3	Mutation > deletion > amplification

MSI, microsatellite instability.

## Data Availability

Not applicable.
